# Abernethy Malformation Type II and Concurrent Nodular Hyperplasia in a Rare Female Case

**DOI:** 10.1155/2018/6501490

**Published:** 2018-07-04

**Authors:** Zhen Kang, Xiangde Min, Liang Wang

**Affiliations:** Department of Radiology, Tongji Hospital, Tongji Medical College, Huazhong University of Science and Technology, Wuhan, Hubei 430030, China

## Abstract

**Background:**

Abernethy malformation is a rare splanchnic vascular abnormality characterizing extrahepatic abnormal shunts that is classified into types I and II. Abernethy malformation type I has a female predilection and is associated with a variety of concurrent hepatic benign or malignant tumours while type II with concurrent tumours is very rare in females.

**Case Report:**

We report a rare female case of Abernethy malformation type II with concurrent occupying lesion in the right liver, which was successfully transplanted; the occupying lesion was pathologically proven to be nodular hyperplasia.

**Conclusion:**

This case might provide further knowledge regarding Abernethy malformation. On imaging, the anatomy of portal vein should be carefully investigated to categorize Abernethy malformation, and a wide variety of differential diagnosis of concurrent occupying lesions should be taken into account.

## 1. Background

Abernethy malformation was first described in 1793, which is characterized by direct drainage of the superior mesenteric vein and splenic vein to the inferior vena cava. The malformation is divided into two types based on the vascular anatomy of the exhepatic shunts. The majority of type I shunts are identified in females, and a variety of benign or malignant tumours may exist simultaneously, whereas type II shunts are almost always recognized in males with a much lower prevalence than type I [1]. Here, we report a rare female case of Abernethy malformation type II with a concurrent mass in the right liver, mimicking a hepatocellular carcinoma or hepatoblastoma on computer tomography (CT), which was ultimately confirmed to be nodular hyperplasia by pathology. This case was successfully transplanted by piggyback method. Through this case report, we might gain further knowledge of Abernethy malformation.

## 2. Case Report

A 10-year-old female visited the emergency room of our hospital with right upper quadrant abdominal pain for 8 days that was not associated with eating. No fever, jaundice, emesis, diarrhea, or melena was reported. Initial blood tests and liver function test revealed no abnormalities other than mild elevation of cytomegalovirus IgG antibody (23.7 U/ml). A routine urine test was positive for red blood cells (3+, 294.8/*μ*l) and white blood cells (3+, 422.9/*μ*l). Alpha-fetoprotein and cancer embryonic antigen were negative, whereas cancer antigen 19-9 was elevated (247.25 U/ml, normal level 2-37 U/ml).

**Figure 1:**
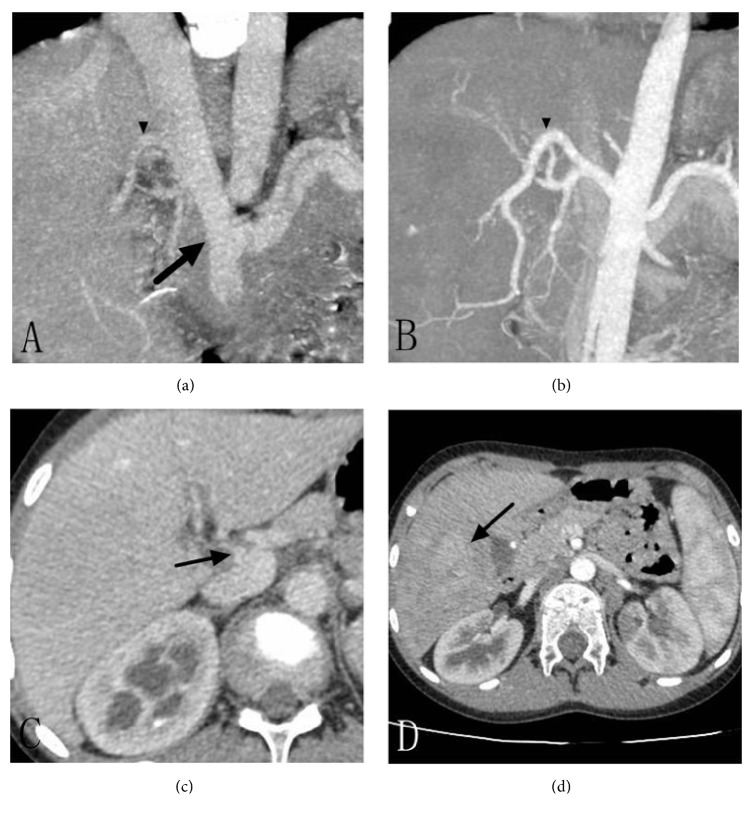
(a) The splenic vein and superior mesenteric vein drained directly into the inferior vena cava after confluence (arrow). (b) Note that the branch overlapping with the portal vein in (a) originated from the hepatic artery, not from the portal vein (arrow head). (c) A barely perceptible small branch of the portal vein measuring only 3 mm supplied the left lobe of the liver (arrow). (d) A faintly visible mass in segment 5 approximately 55 mm in size and poorly demarcated with mildly heterogeneous enhancement on arterial phase (arrow), which conferred a diagnosis of tumour initially.

Ultrasound examination exhibited a mass measuring 78 x 71 mm in the right liver with abundant blood signals. No obvious portal venous trunk and branches were noted in the liver but the superior mesenteric vein and splenic veins drained directly into the inferior vena cava. The left hepatic vein and middle hepatic vein conjoined together and then drained to the inferior vena cava. Abdominal enhanced CT with multiplanar reconstruction revealed that the splenic vein and superior mesenteric vein drained directly into the inferior vena cava after confluence (Figures 1(a) and 1(b)), and only two branches of hepatic veins drained into the inferior vena cava. A barely perceptible small branch of the portal vein measuring 3 mm supplied the left lobe of the liver (Figure 1(c)). In addition, a mass was noted in hepatic segment 5, approximately 55 mm in size and poorly demarcated. Arterial phase of enhancement showed mildly heterogeneous enhancement (Figure 1(d)), while portal venous and delayed phases demonstrated iso- to hypoattenuating. Abernethy malformation (type II) and concurrent hepatocellular carcinoma or hepatoblastoma were suspected.

**Figure 2:**
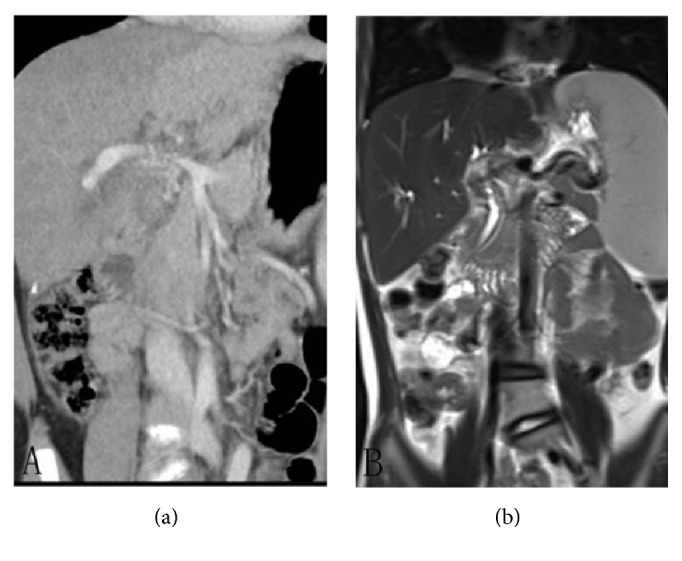
Piggyback liver transplantation was conducted. (a) CT imaging illustrated the reconstructed portal vein was in a satisfactory condition. (b) MR showed the transplanted liver was almost normal in signal 2 years following transplantation.

The liver donor from a close relative was examined comprehensively and the transplantation for this suffered child was conducted 1 month after diagnosis. Surgical findings demonstrated a mass occupying the right liver measuring 60 mm in diameter. No remarkable liver cirrhosis was noted. The superior mesenteric vein and splenic vein merged together and drained directly to the inferior vena cava. A faintly visible branch vessel measuring 3 mm dominated the left lobe of the liver; no veins supplied the left medial lobe and the right lobe of the liver. A left lobe of liver was donated from a close relative. Considering that the donor liver weighed only 250g and the ratio of donor liver to recipient weight was 0.83, an auxiliary piggyback liver transplantation method was conducted. The allogeneic iliac vein was adopted to reconstruct the left branch of the recipient portal vein and the left hepatic vein. Then, the hepatic vein was anastomosed to the recipient vena cava. The right hepatic artery of the recipient and the left lobe of the donor liver were anastomosed with the allogeneic iliac artery. The left hepatic duct, the ductus cysticus, and the biliary tree of the donor liver were also reconstructed by end-to-end anastomosis. Meanwhile, the diseased right lobe of the liver was resected. Two years after the transplantation, physical examination as well as CT and MR imaging showed the patient was in a satisfactory condition (Figures 2(a) and 2(b)); the transplanted liver was practically normal in contour and signal intensity. The child is still alive at present, 4 years after surgery.

**Figure 3:**
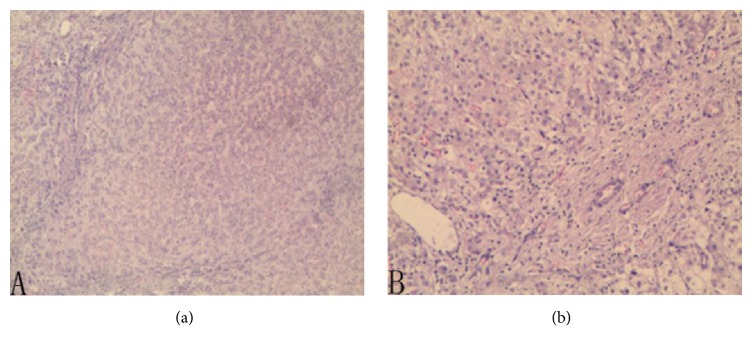
(a, b) Pathologically, the lesion was nodular in appearance and lacked a normal hepatic lobule texture, with absence of normal hepatic veins and bile ducts, while multiple fibrous connective tissues and chronic inflammatory cells scattered over.

Microscopically, the lesion was in nodular appearance without feeding hepatic veins. Abnormal hepatic lobules and distorted bile ducts scattered throughout the mass, with increased amount of fibrous connective tissues and chronic inflammatory cells. Some hepatocytes were characterized by hydropic degeneration, cholestasis, and regeneration (Figures 3(a) and 3(b)). Portal spaces exhibited an absence of veins and were replaced by chronic inflammatory cell infiltration, with 3 lymph nodes featuring reactive hyperplasia. The final pathological diagnosis was congenital extrahepatic portal-systemic shunt and coexisting nodular hyperplasia.

## 3. Discussion

Abernethy malformation was first described in 1793 in an autopsy of a 10-month-old female who died of unknown causes and of whom the portal vein bypassed the liver and directly drained into the inferior vena cava [2]. There have been up to 100 cases currently reported [3]. The abnormalities are believed to result from the persistence of the embryonic vessels [4]. Type I is characterized by the complete drainage of portal blood into the vena cava congenitally and is further divided into type Ia (drainage of the superior mesenteric and splenic veins into systemic veins separately) and type Ib (drainage of the superior mesenteric and splenic veins into systemic veins after they conjoin to form a short extrahepatic portal vein). Type II is rarer and defined as a side-to-side extrahepatic shunt [5]. Abernethy malformation may be complicated by increased serum ammonia, hepatic encephalopathy, or hepatocellular carcinoma in the absence of cirrhosis [6].

Abernethy malformation type I has a female predilection [1] and predisposes patients to develop hepatic tumours and other abnormalities [7]. Histologically, benign tumours, such as focal nodular hyperplasia, nodular regenerative hyperplasia, and liver adenoma, as well as malignant tumours, such as hepatoblastoma and hepatocellular carcinoma, have all been reported [5]. The majority of reported Abernethy type II in publications were males. Here, we reported a female case of Abernethy malformation type II, which is rare in epidemiology and gender, with coincidental huge nodular hyperplasia confirmed pathologically. To our knowledge, such a case has not been reported to date. On imaging, the mass occupied the right lobe of the liver, showing mildly heterogeneous enhancement on arterial phase on contrast-enhanced CT scan, as well as iso to low density on portal venous phase and delayed phase. Considering the huge size of the mass, the age characteristic, and the enhancement features, hepatocellular carcinoma or hepatoblastoma was suspected. However, the final pathology was confirmed as nodular hyperplasia.

In terms of treatment, the ideal management strategy has not yet been established. There have been reports of other treatments, such as shunt ligation [8] and shunt closure using interventional angiography [9]; however, interventional closure may lead to recurrent hyperammonemia as reported [9]. In this case, taking into consideration that the concurrent mass developed in the right liver, piggyback liver transplantation is the optimal treatment.

## 4. Conclusion

This case might provide further knowledge regarding Abernethy malformation. On imaging, the anatomy of portal vein should be carefully investigated to categorize Abernethy malformation, and a wide variety of differential diagnosis of concurrent occupying lesions should be taken into account.
